# Development of quantitative multiplex RT-qPCR one step assay for detection of hepatitis delta virus

**DOI:** 10.1038/s41598-023-37756-z

**Published:** 2023-07-26

**Authors:** Jackson Alves da Silva Queiroz, Tárcio Peixoto Roca, Rutilene Barbosa Souza, Luiz Fellype Alves de Souza, Ana Maísa Passos-Silva, André Luiz Ferreira da Silva, Eugênia de Castro e Silva, Lourdes Maria Pinheiro Borzacov, Rita de Cássia Pontello Rampazzo, Soraya dos Santos Pereira, Thor Oliveira Dantas, Janaína Mazaro, Lívia Melo Villar, Juan Miguel Villalobos Salcedo, Daniel Archimedes da Matta, Deusilene Vieira

**Affiliations:** 1 Fundação Oswaldo Cruz Rondônia FIOCRUZ/RO, Rua da Beira, 7176, Porto Velho, 76812-245 Brazil; 2 Programa de Pós-Graduação em Biologia Experimental, Universidade Federal de Rondônia e Fundação Oswaldo Cruz Rondônia – UNIR/FIOCRUZ/RO, 76801-974 Porto Velho, Brazil; 3 Programa de Pós-Graduação em Medicina Tropical, Instituto Oswaldo Cruz/IOC, FIOCRUZ, 21041-250 Rio de Janeiro, Brazil; 4 Centro de Infectologia Charles Merieux & Laboratório Rodolphe Merieux (FUNDHACRE), Rio Branco, 69918-340 Brazil; 5 Universidade Federal da Bahia – UFBA, Salvador, 40110-909 Brazil; 6 Centro de Pesquisa em Medicina Tropical – CEPEM, Porto Velho, 76812-329 Brazil; 7 Instituto de Biologia Molecular do Paraná - IBMP, 81350-010 Curitiba, Brazil; 8 Universidade Federal do Acre – UFAC, Rio Branco, 69915-900 Brazil; 9 Laboratório Central de Saúde Pública do Acre – LACEN/AC, Rio Branco, 69900-614 Brazil; 10 Universidade Federal de Rondônia - UNIR, Porto Velho, 76801-974 Brazil

**Keywords:** Infectious-disease diagnostics, Virology

## Abstract

Hepatitis Delta is a disease caused by exposure to hepatitis B (HBV) and hepatitis D (HDV) viruses, usually with a more severe clinical outcome when compared to an HBV monoinfection. To date, the real prevalence of HDV infection is underestimated and detection methods are poorly available, especially in more endemic regions. Therefore, a one-step RT-qPCR method for quantification of HDV-RNA was developed. Biological samples were selected between 2017 and 2023 from patients at the Ambulatório Especializado em Hepatites Virais of the Centro de Pesquisa em Medicina Tropical de Rondônia and Serviço de Assistência Especializada and underwent the test developed by this study and a second quantitative RT-qPCR assay. The slope of the initial quantitative assay was − 3.321 with an efficiency of 100.04% and amplification factor equal to 2. Analysis of the repeatability data revealed a Limit of Quantification of 5 copies/reaction and Limit of Detection (95%) of 2.83 copies per reaction. In the diagnostic sensitivity tests, there was an accuracy of 97.37% when compared to the reference test. This assay proved to be highly efficient and reproducible, making it a valuable tool to monitor hepatitis Delta patients and assess the risk of disease progression, as well as the effectiveness of treatment.

## Introduction

The hepatitis delta virus (HDV) classified in the genus Deltavirus is atypical, presenting about 1,700 nucleotides of a single strand of negative polarity RNA with a size between 36 and 43 nm and in its external structure presents surface proteins of the hepatitis B virus (HBV)^1,2^. HDV infection is preceded by previous exposure, current or simultaneously infection with HBV. Usually its clinical outcome will be more severe, characterized by accelerated progression to cirrhosis or hepatocellular carcinoma when compared to HBV monoinfection^3–5^.

It is currently difficult to accurately estimate the prevalence of HDV worldwide; at least 12 to 15 million people may be infected with the virus, distributed across Africa, Asia, the Americas, the Eastern Mediterranean and Europe^6,7^. In South America, the endemic area for HDV is located in the north (Amazon basin region) and covers several countries^5,8–10^. In Brazil, between 2000 and 2021, the number of reported cases was 4,259. These numbers can be considered underreported since this disease remains neglected throughout the country and there is no active search for patients monoinfected with HBV to determine co-infections^11^.

The serological diagnosis of HDV infection is based on the detection of total anti-HDV antibodies in the serum. The presence of HDAg in the hepatic tissue is also a clinical indicator that allows the confirmation of the infection through hepatic elastography, a non-invasive and painless method that allows evaluation of the degree of hepatic fibrosis^4,12^. Once anti-HDV antibodies are detected, molecular diagnostics is used to confirm or rule out active infection. The molecular test is carried out by searching for HDV RNA in plasma, quantified by the RT-qPCR method; however, this is not conventional since there are no kits registered with the Brazilian Health Surveillance Agency (Anvisa). The use of molecular methods to quantify HDV in restricted areas where specialized professionals can develop in-house tests is also a limiting factor^13^.

Therefore, HDV molecular diagnosis remains a challenge because it is not widely available for endemic areas in Amazon region. The aim of the study was to develop a high sensitivity quantitative multiplex one step RT-qPCR assay for the diagnosis and follow-up of patients with Hepatitis Delta.

## Results

### Quantification curve

**Figure 1:**
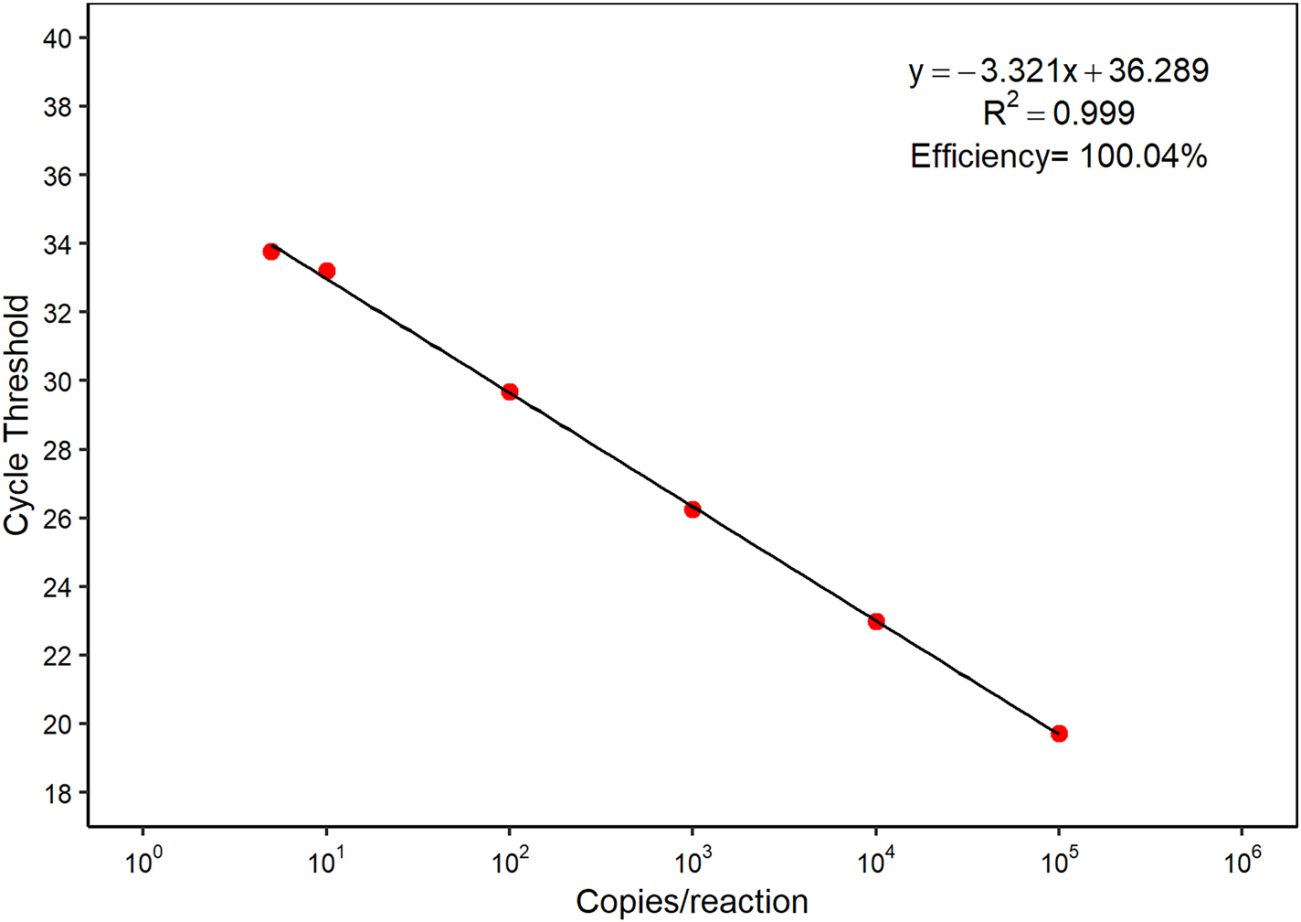
Quantification curve constructed from linear regression analysis of 6 serial dilutions using a recombinant plasmid containing a partial region of the HDV ribozyme, ranging from 5 to 1 × 10^5^ copies per reaction and tested by RT-qPCR; Ct: Cycle Threshold.

The plasmid was tested at intervals of 1 × 10^5^ and 1.25 copies per reaction (1× 10^5^, 1 × 10^4^, 1 × 10^3^, 1 × 10^2^, 1 × 10^1^, 5, 2.5 e 1.25), to determine amplification efficiency and linearity. All points on the quantification curve above 5 copies per reaction had 100% amplification in different assays. The slope of initial quantitative test was − 3.321 with an efficiency of 100.04%, correlation coefficient of the straight line (R2) equal to 0.99, and amplification factor equal to 2 (see Fig. 1).

### Reproducibility and repeatability assays

The 3 assays carried out with the standard dilutions were evaluated individually and collectively, which presented a Coefficient of Variation (CV%) lower than 10% between the tests (Supplementary Table S1). Slope data, line intersection, efficiency and correlation between points are described in Table 1.

**Table 1:** Repeatability and Reproducibility Assays.

Standard (copies/reaction)	1st run (Ct)	2nd run (Ct)	3rd run (Ct)	Mean	SD ( ±)	CV(%)
1 × 10^5^	19,722	19,811	19,853	19,80	0,07	0,34
1 × 10^4^	22,973	23,106	23,128	23,07	0,08	0,36
1 × 10^3^	26,244	26,552	26,382	26,39	0,15	0,58
1 × 10^2^	29,691	29,788	29,872	29,78	0,09	0,30
1 × 10^1^	33,207	33,388	33,431	33,34	0,12	0,36
5 × 10^0^	34,257	34,330	34,324	34,30	0,04	0,12
Slope	− 3,321	− 3,401	− 3,373	–	–	–
Intercept	36,288	36,749	36,639	–	–	–
R^2^	0,999	0,999	0,999	–	–	–
Efficiency	100,04%	96,78%	97,89%	–	–	–

The reproducibility and repeatability assays were performed in three runs on alternate days with dilutions in technical octuplicates, with coefficient of variation (CV%) less than 10% between runs.

### Analytical sensitivity

**Figure 2:**
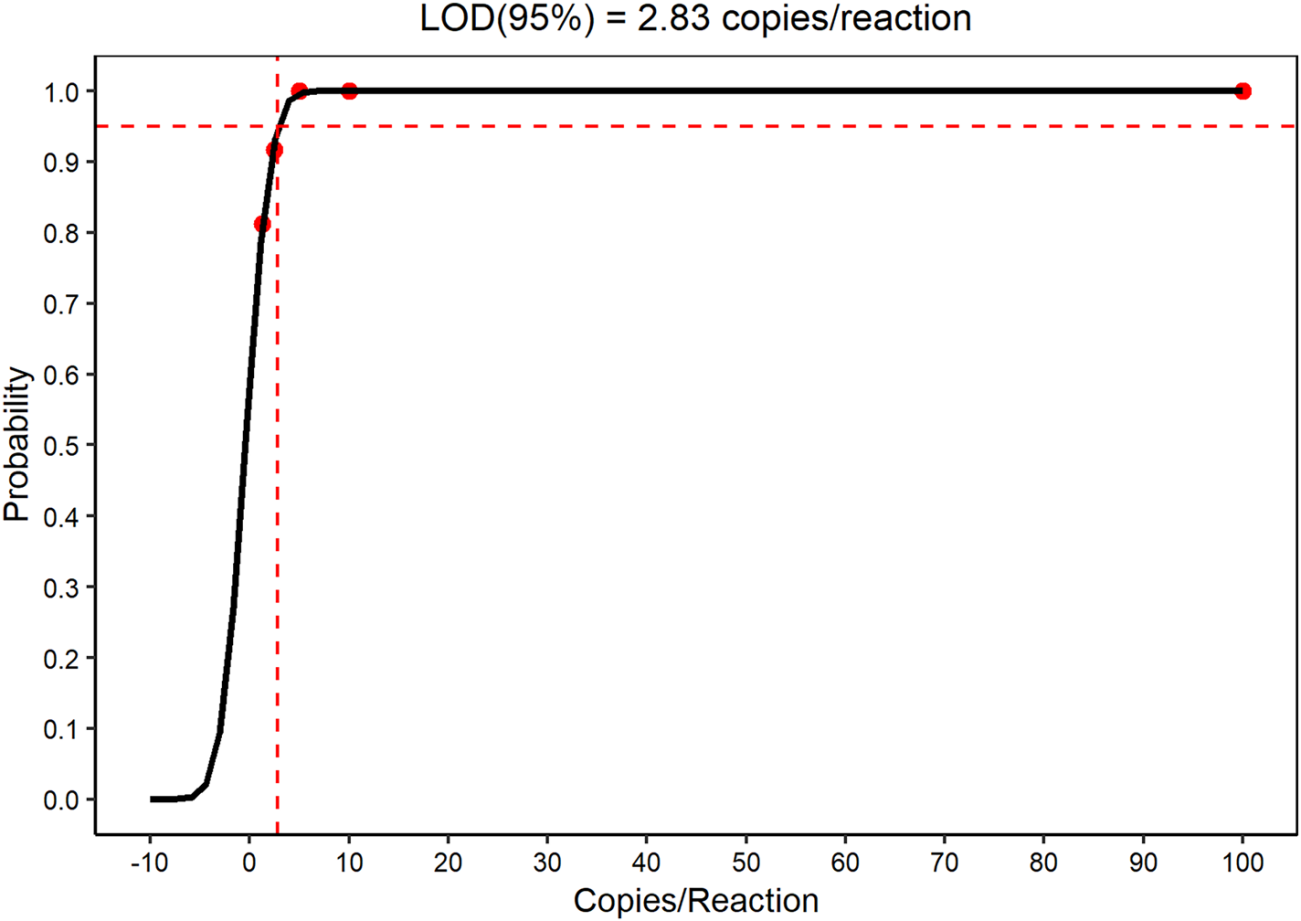
The analysis of replicate dilutions (1 × 10^5^ to 1.25 copies/reaction) revealed a Limit of Quantification (LOQ) of 5 copies per reaction and Limit of Detection (LOD_95%_) of 2.83 copies per reaction for the developed assay. For a more objective visualization, points above 1 × 10^3^ were not included in the figure.

The repeatability data analyzes of dilutions (1 × 10^5^; 1 × 10^4^; 1 × 10^3^; 1 × 10^2^; 1 × 10^1^; 5; 2.5 e 1.25 copies/reaction) revealed a Limit of Quantification (LOQ) of 5 copies per reaction (250 copies/mL) and Limit of Detection (LOD95%) of 2.83 copies per reaction, equivalent to 142 copies/mL of biological samples (see Fig. 2), which is the lowest viral load value that can be detected using the assay developed.

### Diagnostic sensitivity and specificity

**Figure 3:**
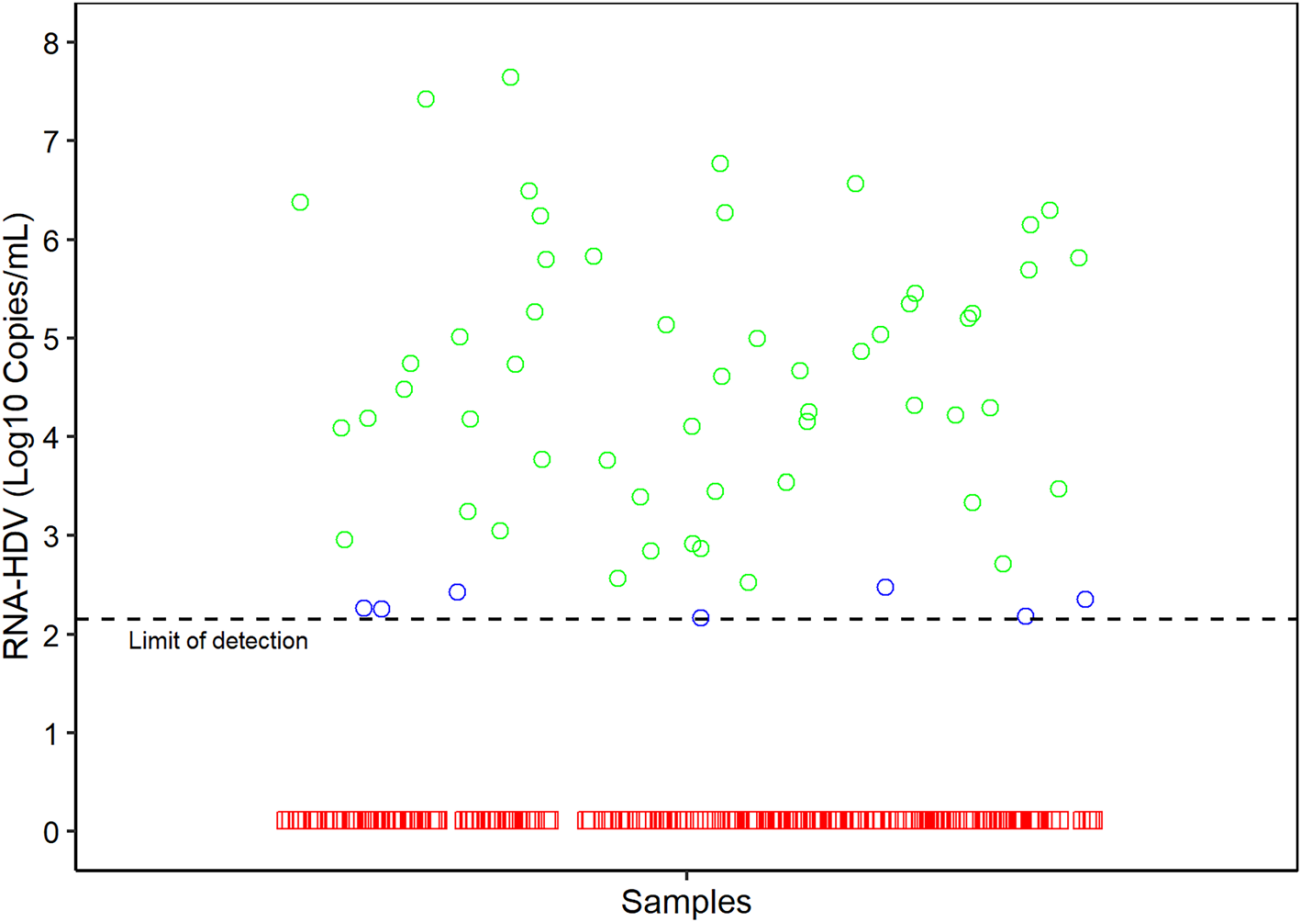
Schematic representation of viral load quantified for 266 samples tested by RT-qPCR for HDV with values in copies/mL (Log10) of biological sample. The dashed line corresponds to the detection limit of the assay. The green circles represent positive samples in both tests and the blue circles represent only positive samples in the test developed in this study. Negative samples are shown as red squares.

Blood samples were collected from 266 patients with chronic infection, positive for anti-HDV, being followed up for Delta hepatitis, of which 16.54% (44/266) were identified as having liver disease and following treatment. Of this total, 64.6% (172/266) were men, aged between 24 and 77 years (mean 45 years; standard deviation (SD) ± 10.23). The samples were tested using the assay developed, where 22.93% (61/266) had a quantifiable viral load, in a range of 2.96 copies per reaction (2.17 Log10 copies/mL) to 9.01 × 10^5^ (7, 65 Log10 copies/mL) of biological sample (see Fig. 3). A total of 65.57% (40/61) of the positive samples have genotyping results for HDV, among which 97.5% (39/40) are characterized as HDV-3 and 2.5% (1/40) as HDV-1 (Supplementary Table S4).

A total of 11.47% (7/61) of positive samples were negative for the reference test^14^, which corresponds to 2.63% (7/266) of samples tested. When compared to the reference test, the development assay showed 100% (95% CI: 93.40–100) sensitivity, 96.70% (95% CI: 93.32–98.66) specificity, 88, 52% for Positive Predictive Value (95% CI: 77.78–95.26) and 100% (95% CI: 98.22–100) for Negative Predictive Value, with an accuracy of 97.37% (95% CI: 94. 65–98.94). The inter-rater agreement quantified by the Kappa (K) statistic was 0.92 (95%CI: 0.86–0.98), which shows a good strength of agreement between the tests. The ROC curve was constructed and the Area Under the Curve (AUC) was calculated to be 1.00 (Supplementary Figure S2). The divergent samples had a viral load of less than 7 copies per reaction (2.96–6.07 copies/reaction), which may explain the disagreement in the assay results and low viral load. All samples negative for HDV and positive for other viruses (DENV, SARS-CoV, and HBV monoinfected, N = 26) showed negative results when subjected to the current test.

## Discussion

Hepatitis delta is a public health problem in endemic areas in South America. This is a neglected disease that affects vulnerable populations, such as indigenous and riverside dwellers in the Amazon basin^5^.

In general, there is a global lack of data to understand epidemiology and clinical features. There are two distinct forms of transmission, the so-called co-infection, which is characterized by the simultaneous transmission of HBV and HDV, or even superinfection, when HDV transmission is preceded by the chronic form of HBV^4,15^.

In this study, an assay was developed to quantify HDV RNA viral load different with high sensitivity and efficiency. The one step RT-qPCR technique developed in the present study showed good efficiency and reproducibility, which is significant for greater precision in quantification from a standard curve.

The methodology used was based on procedures described in the literature and respects quantitative international standards for molecular diagnosis, where the alignment of parameters for carrying out RT-qPCR assays is essential to reduce the risk of false positives and inconclusive results, which decreases assay reliability^16,17^. The assay meets the established criteria for validating a quantitative qPCR, such as efficiency, linear dynamic range, Limit of Detection (LOD) and accuracy, which ensures its robustness^16^.

The efficiency estimated from the slope showed values close to 100% and an amplification factor equal to 2, in accordance with guidelines for standardization of tests that consider a high-performance test with values between 90 and 110%^16,18^. Likewise, the linear dynamic range was determined with 5 different concentrations (Log10), with a minimum of 3 being recommended^16,19,20^, in addition to intra-assay (repeatability) and inter-assay (reproducibility) precision expressed by Standard Deviation (SD) measurements of replicates on a standard curve^16,21,22^.

The LOD was defined as the lowest value tested with 5% of failed reactions, which demonstrated a limit of 142 copies per mL, being similar to the 130 copies/mL obtained in our previous study; however in the current assay, in addition to the addition of an Endogenous control for monitoring reaction quality, the assay allows Reverse Transcription and qPCR to be performed in a single reaction^23^. In comparison, other assays for quantification of HDV-RNA showed a limit of detection that ranged from 15 to 750 copies/mL^24–26^.

During the experiments on patient samples, an improvement in the quantification of HDV-RNA was observed by applying the heat shock step before the Reverse Transcription step. The same result was observed in a study that evaluated the effect of shock in serially diluted clinical samples^27^. This could be explained by the nature of the HDV genome, which has a high G/C ratio and which makes internal base pairing ~ 70%^2^.

In this study, we chose to use a recombinant plasmid as the assay quantification standard that presented a consensus sequence of the 8 HDV genotypes, bearing in mind that the Amazon region presents a predominance of Genotype 3^10,28,29^, and the standard established by the WHO for HDV quantification is derived from genotype 1^30^. Likewise, the primers used in the standardization were designed in order to detect a conserved region for the 8 virus genotypes, guaranteeing the sensitivity and specificity of the test. It is important to point out that despite its practical application, the pattern constructed with a partial HDV genome has its limitations because it is not a real biological sample, however, we used the plasmid diluted in a matrix of nucleic acids extracted from a negative sample to HDV to mimic a biological sample.

The results of 7 samples that disagreed between the current test and reference test can be explained by the borderline values for quantification, however, the comparison was limited only to qualitative results because there are different units of measure for quantification. Furthermore, the possibility of possible false positives is ruled out by the fact that all patients included in this analysis are positive for serology (Anti-HDV). Additionally, most of patients in this study have received therapy for hepatitis Delta, which justifies the fact that despite the positive serology for HDV, not all of them had a quantifiable viral load, as observed in both assays performed.

When it comes to HDV detection, diagnosis can be made both by detecting anti-HDV antibodies and by searching for direct markers, such as the HDV antigen, and by detecting the circulating viral genome^15,31^. One of the main limitations of diagnosing HDV using serological method is the failure to elucidate the current infection, being limited to a marker of contact with the virus, which is different from what occurs for HBV, where it is possible to determine the different stages of that disease^32^. In this case, the use of a quantitative assay can help in understanding the different stages of HDV infection.

Serological diagnosis is not widely applied in Brazil, which makes it difficult to know the real prevalence of HDV in this country. It is important to raise awareness and efforts by health authorities and population for the importance of serological diagnosis. According to a recommendation of a guideline to Diagnosis of Viral Hepatitis in Brazil, hepatitis Delta should be investigated in individuals who present reactive results in immunoassays for HBsAg and who reside or have been in endemic areas for this condition^33^.

According to WHO, there is still a limitation in the availability of HDV diagnosis and absence of direct HDV RNA detection methods, which are normally used to monitor the response to antiviral therapy^34^. As with HBV and HIV, the standardization of laboratory diagnosis by Real-Time PCR allowed significant advances in the clinical protocol and therapeutic guidelines for adequate treatment and control, with more specific approaches aimed at patients^35^. The expansion of diagnostic coverage for HDV in Brazil would enable the elaboration of guidelines for specific treatment and the diagnostic algorithm (for identification in suspected cases), reducing the risk of evolution, with remission of acute and chronic liver activities (decrease of manifestations and necroinflammatory activities, cirrhosis and hepatocellular carcinoma)^36^.

In this early decade, researchers still addressed issues to establishing a satisfactory treatment for those patients infected with HDV chronic, even though the current therapy with Peg-IFN (pegylated interferon) is dated with approximately 35 years of medical availability^32,37^. The existing obstacles in relation to the effectiveness of the treatment for HDV are associated with the direct dependence on the prescription of therapies aimed at the treatment of HBV in infected individuals. Thus, the therapeutic approach is conditioned to circulating HBsAg levels, based on international guidelines, which direct the management of medications in individuals with HDV only when, in clinical follow-up, they present a viral load for HBV > 2,000 IU/mL^38,39^. In this case, the application of a quantitative RT-qPCR assay for HDV detection will allow identifying, quantifying, and monitoring viral replication during the course of the disease in patients.

In addition to an accurate serological and quantitative molecular diagnosis for HDV, it is necessary to raise the awareness of health agencies and governments on this issue, with special attention to countries with a high number of confirmed cases. Vaccine Programs to HBV, but also mapping HDV endemic areas, tracking cases and providing properly treatment to HDV cases could be useful to prevent future transmission.

In conclusion, Tthis current assay has demonstrated high efficiency and reproducibility, making it a valuable tool to monitor HDV carriers and assess disease progression as well as treatment efficacy. In addition, this assay may allow studies to link viral load levels and persistence of chronic HDV, what brings higher risk of developing cirrhosis and hepatocellular carcinoma.

## Methods

### Ethics declarations

This study was approved by the research ethical committee from Centro de Pesquisa em Medicina Tropical de Rondônia – CEPEM/RO (Nº 3.826.726), and informed consent was obtained from all participants. The research procedures were carried out according to the ethical principles stipulated by the 1975 World Medical Assembly and the Brazilian Ministry of Health and follows the norms established in Resolution No. 466, December 12, 2012.

### Study site and biological samples

Cross-sectional research was developed at Laboratório de Virologia Molecular da Fundação Oswaldo Cruz Rondônia – FIOCRUZ/RO and Centro de Infectologia Charles Merieux & Laboratório Rodolphe Merieux (FUNDHACRE). Blood samples from patients were collected in the period between 2017 and 2023 at the Ambulatório Especializado em Hepatites Virais at CEPEM-RO and Serviço de Assistência Especializada (SAE-SESACRE). Two groups of patients were screened: HDV carriers with previous diagnosis by ELISA (Positive to Anti-HDV, HBsAg and Anti-HBc-Total; n = 266); and a second group infected by some other viruses (n = 26), HBV (16/26), DENV (5/26) and SARS-CoV-2 (5/26) with no evidence of HDV infection (Negative to Anti-HDV). Clinical and laboratory exam information were collected from medical records.

### Viral RNA extraction

The nucleic acid was extracted using magnetic beads method followed by an automated extractor EXTRACTA 32 DNA and RNA extractor (LOCCUS, São Paulo, Brazil), and purifier with EXTRACTA KIT FAST – Viral DNA/RNA (MVXA-P016FAST) according to manufacturer's instructions and eluted in 50 µL of Elution Buffer.

### Quantification standard curve

To construct the quantification standard, reference sequences for the different HDV genotypes deposited in the Genbank of the National Center for Biotechnology Information (NCBI) were selected. The sequences were aligned using the ClustalW algorithm (Molecular Evolutionary Genetics Analysis – MEGA, Version 11)^40^. After alignment, a consensus sequence of 1014 base pairs (bp) corresponding to all 8 genotypes was constructed. The selected region partially comprises the complete HDV genome (initial position 675 and final 1682; NCBI Reference Sequence: NC_001653.2) comprising the short, long, ribozyme and non-coding region (NTR) regions of the viral antigen (HDVAg). The insert was commercially synthesized and cloned into pUC57 vector (Genscript Biotech Corporation, New Jersey, USA) generating a recombinant plasmid with a size of 3724 base pairs (Supplementary Figure S3).

The plasmid concentration in ng/μL was determined by fluorimetry using the Qubit 4 dsDNA High Sensitivity kit (ThermoFisher Scientific®, Massachusetts, USA) and the initial copy number of 2.12 × 10^8^ was calculated based on the recombinant DNA size and molecular weight, according to the previously described method^41^, based on the following Eq. (1):

1 $$copy \;number = \left[ {\frac{plasmid\,concentration\,ng/\mu L}{{plasmid\,bp\,number + insert\,bp\,number}} \times 650} \right] \times avogadro^{\prime}s\,constant \left( {6,02\,x\,10^{23} } \right)$$

The recombinant plasmid was serially diluted into a biological matrix of nucleic acids extracted from hepatitis Delta negative human serum at the following concentrations, 1 × 10^4^; 1 × 10^3^; 1 × 10^2^; 1 × 10^1^; 1 × 10^0^; 0.5 e 0.125 copies per microliter to simultaneously amplify the viral target (HDV) and the human endogenous control (RNase P).

### Quantitative one-step multiplex RT-qPCR assay

To quantify HDV-RNA, primers and probes were designed to detect the Ribozyme region, which is highly conserved^42^, as seen in Supplementary Figure S3. To disrupt secondary structures of HDV genome, genetic material extracted from biological samples was incubated at 95 °C for 5 min followed by ice for 1 min. The multiplex reaction was standardized using 5 µL of TaqMan Fast Virus 1-Step Master Mix (Applied Biosystems®, California, USA), 10 µL of each curve dilution or genetic material from biological sample, 5 µL of oligomix containing 300 nM of each primer and 100 nM for the HDV probe, and 500 nM of each primer and 125 nM of probe for detection of Endogenous Control (Table 2).

**Table 2:** One-Step Multiplex RT-qPCR primers and probes.

Target gene	Name	Description	Sequence 5'-3'	Amplicon (bp)
Ribozyme	HDVqF	Sense	TGGCTCTCCCTTAGCCATCCGA	86
	HDVqR	Antisense	GGTCGGCATGGSATCTCCACC	
	HDVqP	Probe	FAM—CGGATGCCCAGGTCGGACC – MGB (applied biosystems)	
RNase P	RP-F	Sense	AGATTTGGACCTGCGAGCG	65
	RP-R	Antisense	GAGCGGCTGTCTCCACAAGT	
	RP-P	Probe	HEX—TTCTGACCTGAAGGCTCTGCGCG—BHQ1 (LGC biosearch technologies)	

Oligonucleotides for HDV RNA detection were designed for this study. For detection of human RNase P, oligonucleotides adapted from Centers for Disease Control and Prevention (CDC) were used^43^.

The reaction was performed on a QuantStudio™ 7 Pro Real Time PCR System under following conditions: 50 °C for 30 min for reverse transcription and 95 °C for 15 min for PCR activation followed by 40 cycles of 95 °C for 15 s and 63 °C for 30 s, where the fluorescence was captured. Data were analyzed in Software Design & Analysis Version: 2.6.0 using a baseline of 3–15 and threshold 0.1 for both targets.

### Assay performance analysis

Reproducibility and repeatability were measured through 3 tests performed on consecutive days by different operators. The points with 1 × 10^5^; 1 × 10^4^; 1 × 10^3^; 1 × 10^2^; 1 × 10^1^; 5; 2.5 and 1.25 copies/reaction were used in technical octuplicates for each day. Analytical sensitivity was determined using repeatability data, which served as the basis for measuring the Limit of Detection (LOD95%).

### Diagnostic sensitivity and specificity

To determine the diagnostic sensitivity, the biological samples were submitted to test developed to HDV RNA quantification. All tested samples were subjected to a second commercial quantitative RT-qPCR test (RealStar® HDV RT-PCR Kit 1.0; Altona Diagnostics, Hamburg, Germany) as a reference^14^ and compared to the data obtained. Positive samples for other viruses and negative for HDV (DENV, SARS-CoV, and monoinfected HBV) were tested using the assay developed to estimate diagnostic specificity.

### Statistical analysis

Descriptive analyses were represented through frequencies, central tendency, and dispersion. The Standard Curve was performed using R v4.2.1^44^ software and to calculate the Limit of Detection (LOD95%), the analytical sensitivity data were subjected to binomial regression analysis using the Probit statistical model. The diagnostic sensitivity and specificity results were used to estimate the values of sensitivity, specificity, Positive Predictive Value, Negative Predictive Value, Kappa Agreement Index and construction of the ROC curve, using reference assay^14^.

## Supplementary Information

Supplementary Information.

## Data Availability

The datasets generated during and/or analysed during the current study are available from the corresponding author on reasonable request.
