# Supplementary material for: Development of quantitative multiplex RT-qPCR one step assay for detection of hepatitis delta virus
Source: Sci Rep. 2023 Jul 26;13:12073. doi: 10.1038/s41598-023-37756-z (PMC10372040; doi:10.1038/s41598-023-37756-z)
Supplement: Supplementary file 1 — Supplementary Information. [file 41598_2023_37756_MOESM1_ESM.docx]

**Development of quantitative multiplex RT-qPCR one step assay for detection of Hepatitis Delta Virus**

**Jackson Alves da Silva Queiroz^1,2,+^, Tárcio Peixoto Roca^1,3,+^, Rutilene Barbosa Souza^4,5^, Luiz Fellype Alves de Souza^3,4^, Ana Maísa Passos-Silva^1,2^, André Luiz Ferreira da Silva^1,2^, Eugênia de Castro e Silva^6^, Lourdes Maria Pinheiro Borzacov^6^, Rita de Cássia Pontello Rampazzo^7^, Soraya dos Santos Pereira^1,2^, Thor Oliveira Dantas^8^, Janaína Mazaro^9^, Lívia Melo Villar^3^, Juan Miguel Villalobos Salcedo^10^, Daniel Archimedes da Matta^4^, and Deusilene Vieira^1,2*^**

|  | **Standard (copies/reaction)** | | | | | | | |
| --- | --- | --- | --- | --- | --- | --- | --- | --- |
|  | **1x10^5^** | **1x10^4^** | **1x10^3^** | **1x10^2^** | **1x10^1^** | **5** | **2.5** | **1.25** |
| **1st run (Ct)** | 19,807 | 22,964 | 26,322 | 29,891 | 33,314 | 34,572 | 36,012 | 36,059 |
|  | 19,656 | 23,006 | 26,295 | 29,824 | 34,122 | 33,686 | Und* | 37,778 |
|  | 19,665 | 22,996 | 26,468 | 29,625 | 33,089 | 33,648 | 34,156 | 35,963 |
|  | 19,668 | 22,950 | 26,414 | 29,567 | 32,424 | 33,462 | 35,888 | 35,996 |
|  | 19,702 | 23,029 | 26,333 | 29,434 | 33,211 | 34,316 | 34,930 | Und* |
|  | 19,772 | 23,010 | 26,373 | 29,664 | 33,089 | 33,203 | 35,153 | 36,939 |
|  | 19,808 | 22,854 | 25,436 | 29,899 | 33,034 | 33,300 | 37,751 | 36,638 |
|  | 19,696 | 22,976 | 26,312 | 29,621 | 33,371 | 33,909 | 36,004 | 36,357 |
| **Mean** | **19,72** | **22,97** | **26,24** | **29,69** | **33,21** | **33,76** | **35,70** | **36,53** |
| **SD (±)** | **0,06** | **0,05** | **0,33** | **0,17** | **0,47** | **0,48** | **1,13** | **0,66** |
| **CV(%)** | **0,32** | **0,24** | **1,26** | **0,56** | **1,42** | **1,42** | **3,17** | **1,80** |
| **2nd run (Ct)** | 19,796 | 23,043 | 26,678 | 29,952 | 33,491 | 34,944 | 35,648 | 35,435 |
|  | 19,845 | 23,218 | 26,608 | 30,071 | 33,891 | 34,268 | 34,816 | 35,347 |
|  | 19,825 | 23,143 | 26,897 | 29,493 | 32,774 | 34,691 | 34,986 | 39,002 |
|  | 19,786 | 22,967 | 26,825 | 30,117 | 32,877 | 34,220 | 34,281 | Und* |
|  | 19,817 | 23,063 | 26,741 | 29,699 | 33,087 | 35,267 | 35,411 | 35,325 |
|  | 19,783 | 23,147 | 26,429 | 29,577 | 33,603 | 33,505 | Und* | 35,186 |
|  | 19,806 | 23,155 | 25,986 | 29,700 | 33,641 | 35,201 | 35,501 | 36,718 |
|  | 19,827 | 23,115 | 26,257 | 29,692 | 33,743 | 33,520 | 34,763 | 34,742 |
| **Mean** | **19,81** | **23,11** | **26,55** | **29,79** | **33,39** | **34,45** | **35,06** | **35,97** |
| **SD (±)** | **0,02** | **0,08** | **0,31** | **0,23** | **0,42** | **0,69** | **0,49** | **1,47** |
| **CV(%)** | **0,11** | **0,34** | **1,17** | **0,77** | **1,25** | **2,02** | **1,39** | **4,09** |
| **3rd run (Ct)** | 19,855 | 23,233 | 26,632 | 29,929 | 33,831 | 34,397 | 37,228 | 37,712 |
|  | 19,803 | 23,089 | 26,327 | 29,809 | 33,974 | 33,863 | 34,959 | Und* |
|  | 19,911 | 23,114 | 26,232 | 29,989 | 33,049 | 34,374 | 34,882 | 34,642 |
|  | 19,798 | 23,069 | 26,398 | 29,809 | 34,437 | 34,004 | 34,611 | 36,327 |
|  | 19,866 | 23,167 | 26,403 | 29,556 | 33,423 | 33,754 | 35,180 | 36,343 |
|  | 19,819 | 23,179 | 26,508 | 30,064 | 32,936 | 33,649 | 35,334 | 37,369 |
|  | 19,899 | 22,999 | 26,145 | 29,848 | 33,170 | 34,815 | 36,425 | 35,104 |
|  | 19,874 | 23,173 | 26,412 | 29,972 | 32,627 | 34,815 | 34,839 | Und* |
| **Mean** | **19,85** | **23,13** | **26,38** | **29,87** | **33,43** | **34,21** | **35,43** | **36,25** |
| **SD (±)** | **0,04** | **0,07** | **0,15** | **0,16** | **0,61** | **0,46** | **0,91** | **1,21** |
| **CV(%)** | **0,22** | **0,32** | **0,58** | **0,53** | **1,81** | **1,34** | **2,58** | **3,33** |

**Supplementary Table S1** - Cycle Threshold (Ct) values obtained for each dilution of the quantification standard (copies/reaction) in reproducibility and repeatability assays. The tests were carried out on 3 consecutive days with different operators, each test being performed in technical octuplicates. *Und = Undetermined (Ct value not detected).

**
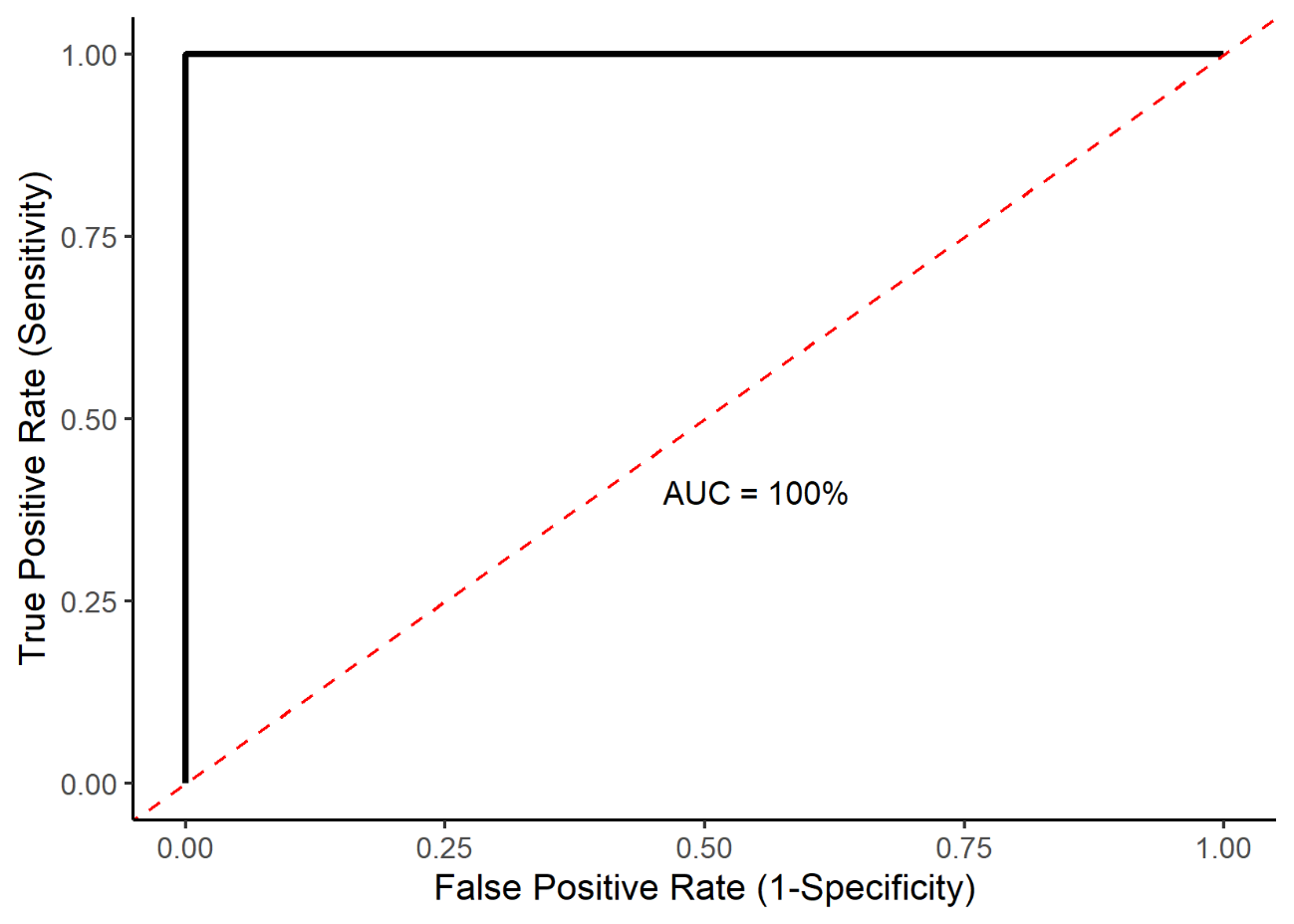
Supplementary Figure S2** – ROC curve constructed from the viral load values in Log10 copies/mL of each patient quantified using the developed test and compared with the result obtained with the reference test. The Area Under the Curve (AUC) was calculated to be 1.00.


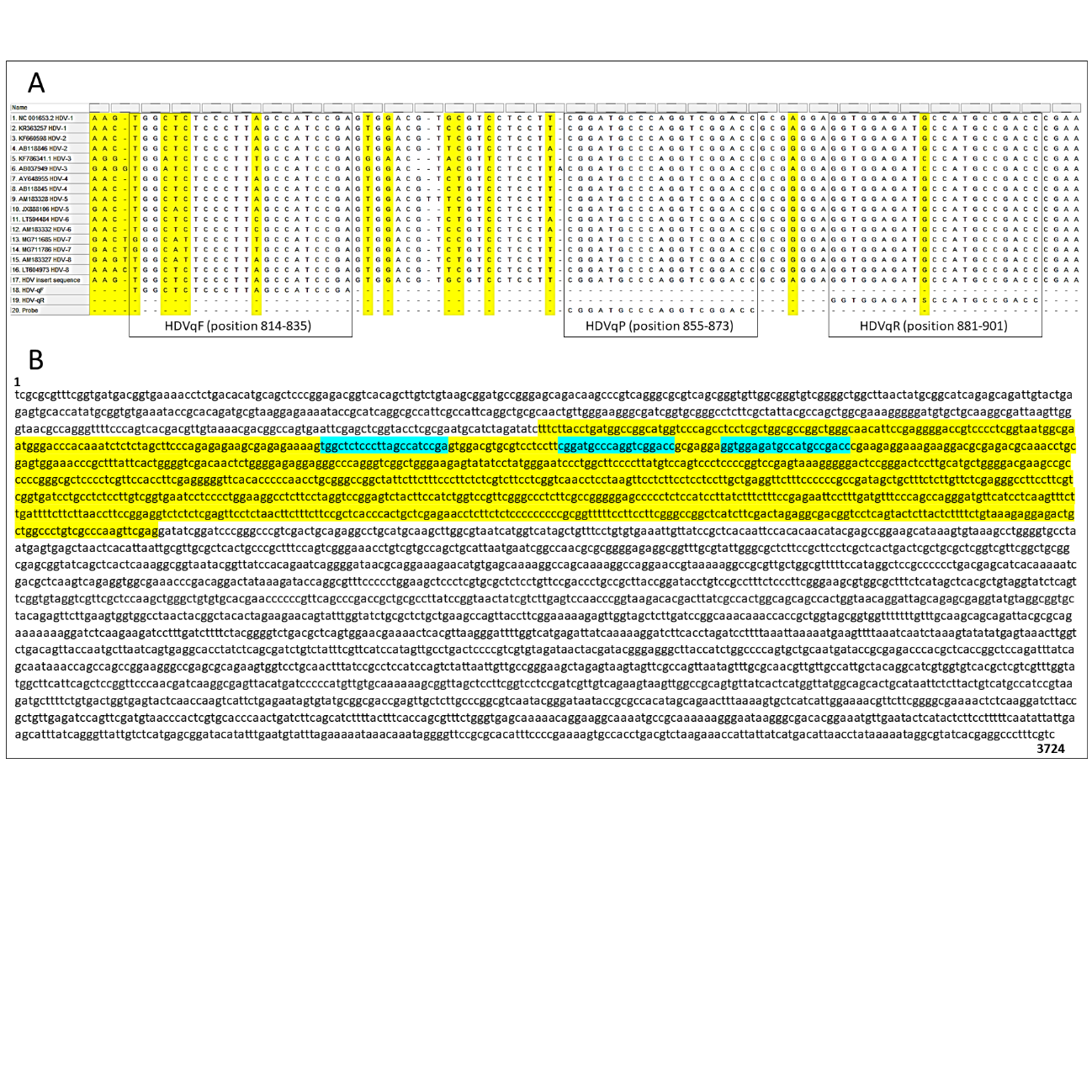


**Supplementary Figure S3 –** (A) Alignment of primers and probes with sequences of the 8 HDV genotypes and the insert of the HDV genome used for construction of the recombinant plasmid. The starting and ending positions of the oligonucleotides were measured based on the reference sequence (NCBI Reference Sequence: NC_001653.2). Highlighted sites correspond to variable nucleotides between genotypes. (B) Complete sequence of the recombinant plasmid (3724 bp) containing the HDV insert (yellow) and the detection region of primers and probes (cyan). Unmarked regions correspond to the pUC57 cloning vector sequence.

| **Sample**  **( N=40)** | **HDV Genotype** | **Viral Load**  **(LOG10 copies/mL)** | **Gender** | **Age (years)** | **Collection city** | **Total anti-HDV ELISA (IgM/IgG)** |
| --- | --- | --- | --- | --- | --- | --- |
| 1 | HDV-1 | 2,38 | Male | 55 | Porto Velho/RO/Brazil | (+) |
| 2 | HDV-3 | 2,84 | Male | 77 | Porto Velho/RO/Brazil | (+) |
| 3 | HDV-3 | 4,67 | Male | 69 | Porto Velho/RO/Brazil | (+) |
| 4 | HDV-3 | 5,14 | Male | 47 | Porto Velho/RO/Brazil | (+) |
| 5 | HDV-3 | 5,80 | Male | 47 | Porto Velho/RO/Brazil | (+) |
| 6 | HDV-3 | 4,09 | Male | 42 | Porto Velho/RO/Brazil | (+) |
| 7 | HDV-3 | 5,46 | Female | 41 | Porto Velho/RO/Brazil | (+) |
| 8 | HDV-3 | 6,24 | Female | 40 | Porto Velho/RO/Brazil | (+) |
| 9 | HDV-3 | 5,25 | Male | 40 | Porto Velho/RO/Brazil | (+) |
| 10 | HDV-3 | 4,87 | Male | 33 | Porto Velho/RO/Brazil | (+) |
| 11 | HDV-3 | 5,35 | Female | 32 | Porto Velho/RO/Brazil | (+) |
| 12 | HDV-3 | 6,57 | Male | 50 | Porto Velho/RO/Brazil | (+) |
| 13 | HDV-3 | 4,74 | Female | 81 | Porto Velho/RO/Brazil | (+) |
| 14 | HDV-3 | 2,87 | Male | 60 | Porto Velho/RO/Brazil | (+) |
| 15 | HDV-3 | 3,77 | Male | 51 | Porto Velho/RO/Brazil | (+) |
| 16 | HDV-3 | 6,78 | Male | 42 | Porto Velho/RO/Brazil | (+) |
| 17 | HDV-3 | 4,32 | Male | 49 | Porto Velho/RO/Brazil | (+) |
| 18 | HDV-3 | 4,19 | Male | 46 | Porto Velho/RO/Brazil | (+) |
| 19 | HDV-3 | 4,48 | Female | 50 | Porto Velho/RO/Brazil | (+) |
| 20 | HDV-3 | 6,38 | Male | 53 | Porto Velho/RO/Brazil | (+) |
| 21 | HDV-3 | 6,30 | Male | 59 | Porto Velho/RO/Brazil | (+) |
| 22 | HDV-3 | 4,11 | Male | 37 | Porto Velho/RO/Brazil | (+) |
| 23 | HDV-3 | 7,39 | Female | 71 | Rio Branco/AC/Brazil | (+) |
| 24 | HDV-3 | 5,04 | Male | 58 | Rio Branco/AC/Brazil | (+) |
| 25 | HDV-3 | 4,86 | Female | 48 | Rio Branco/AC/Brazil | (+) |
| 26 | HDV-3 | 5,82 | Female | 47 | Rio Branco/AC/Brazil | (+) |
| 27 | HDV-3 | 5,91 | Female | 46 | Rio Branco/AC/Brazil | (+) |
| 28 | HDV-3 | 4,32 | Female | 46 | Rio Branco/AC/Brazil | (+) |
| 29 | HDV-3 | 4,75 | Female | 46 | Rio Branco/AC/Brazil | (+) |
| 30 | HDV-3 | 5,89 | Female | 42 | Rio Branco/AC/Brazil | (+) |
| 31 | HDV-3 | 7,38 | Female | 42 | Rio Branco/AC/Brazil | (+) |
| 32 | HDV-3 | 6,30 | Female | 39 | Rio Branco/AC/Brazil | (+) |
| 33 | HDV-3 | 6,27 | Female | 37 | Rio Branco/AC/Brazil | (+) |
| 34 | HDV-3 | 5,29 | Female | 32 | Rio Branco/AC/Brazil | (+) |
| 35 | HDV-3 | 4,47 | Male | 30 | Rio Branco/AC/Brazil | (+) |
| 36 | HDV-3 | 5,50 | Female | 29 | Rio Branco/AC/Brazil | (+) |
| 37 | HDV-3 | 4,63 | Male | 29 | Rio Branco/AC/Brazil | (+) |
| 38 | HDV-3 | 3,98 | Male | 28 | Rio Branco/AC/Brazil | (+) |
| 39 | HDV-3 | 4,26 | Male | 28 | Rio Branco/AC/Brazil | (+) |
| 40 | HDV-3 | 4,82 | Male | 21 | Rio Branco/AC/Brazil | (+) |
| **Mean** | **-** | **5,09** | **-** | **45,50** | **-** | **-** |
| **SD (±)** | **-** | **1,16** | **-** | **13,48** | **-** | **-** |
| **CV(%)** | **-** | **22,75** | **-** | **29,62** | **-** | **-** |

**Supplementary Table S4 -** Epidemiological data and viral load of HDV samples with genotyping results
